# Dechlorane Plus and Related Compounds in Food—A Review

**DOI:** 10.3390/ijerph18020690

**Published:** 2021-01-14

**Authors:** Elisa Ghelli, Ronan Cariou, Gaud Dervilly, Giampiero Pagliuca, Teresa Gazzotti

**Affiliations:** 1 Department of Veterinary Medical Sciences, University of Bologna, Ozzano dell’Emilia, 40064 Bologna, Italy; elisa.ghelli2@unibo.it (E.G.); giampiero.pagliuca@unibo.it (G.P.); 2 LABERCA (Laboratoire d’Etude des Résidus et Contaminants dans les Aliments), Oniris, INRAE (Institut National de Recherche Pour L’agriculture, L’alimentation et L’environnement), F-44307 Nantes, France; ronan.cariou@oniris-nantes.fr (R.C.); gaud.dervilly@oniris-nantes.fr (G.D.); 3 Health Sciences and Technologies-Interdepartmental Center for Industrial Research (CIRI-SDV), University of Bologna, Ozzano dell’Emilia, 40064 Bologna, Italy

**Keywords:** dechlorane-related compounds, persistent organic pollutant, foodstuff, environmental contaminant, food analysis

## Abstract

Dechlorane Plus is a polychlorinated compound which has exclusively anthropic origin. This compound has been manufactured for close to 60 years for various applications, but mainly as flame retardant. Dechlorane Plus and other Dechlorane-related compounds (DRCs) are currently marketed as a replacement for Dechlorane, also known as Mirex, banned in 1978. These compounds share comparable properties to persistent organic pollutants (POPs), such as persistence in the environment, high lipophilicity, bioaccumulation through the food web and adverse effects on the environment and human health. Despite their long production history, they have been only recently reported in various environmental compartments, such as air, soil, and foodstuff. The aim of this review is to provide a picture of the current state of knowledge on worldwide DRC levels in food, in order to highlight gaps and research needs. The review compares the data on DRC contamination available in literature, considering different food categories and sampling country. In addition, it is specified whether the data were obtained from studies on foodstuff to estimate dietary intake, to evaluate the contamination near the e-waste treatment area or for environmental monitoring purposes.

## 1. Introduction

Dechlorane, also known as Mirex, was widely marketed as a pesticide as well as a flame retardant (FR) in the USA from the 1960s to the 1970s [1]. FRs are a wide range of chemicals generally used in manufacture of electronic, textiles, plastics and building materials in order to inhibit the development and propagation of flames, increasing the safety of these products [2]. There are different groups of FRs based on their chemical characteristics. One of these is represented by halogen-containing compounds which includes Dechlorane-related-compounds (DRCs). Due to its toxicity, persistence and high bioaccumulation potential, Mirex was banned in the United States in 1978 [3], consequently, other related compounds such as Dechlorane Plus (DP), Dechlorane 601 (Dec-601), Dechlorane 602 (Dec-602), Dechlorane 603 (Dec-603), Dechlorane 604 (Dec-604) and Chlordene Plus (CP), patented by the former Hooker Chemicals and Plastics Corp. (Hooker; currently OxyChem, Niagara Falls, NY, USA), were developed for replacing Mirex [1].Table 1 shows the compounds’ name, molecular and the structural formula and the two abbreviations that have been used in the literature: the first is the one most commonly found in the considered works, the second is the official abbreviations established by the scientific community in 2012 [4].

The commercially available formulation of DP contains two stereoisomers, *syn*-DP (CAS# 135821-03-3) and *anti*-DP (CAS# 135821-74-8), in the approximate ratio of 1:3 so the *anti*-isomer represents 75% of the total [5]. All these compounds (see Table 1) are highly chlorinated and share a bicyclo(2,2,1)heptene structure, resulting from a Diels–Alder reaction between one or two hexachlorocyclopentadiene molecules and various cyclic dienophiles [1]. DP is poorly soluble in water and is extremely lipophilic, having a very high octanol-water partition coefficient very high (Log Kow = 9.3). DP is classified as a low production volume chemical in EU, while it is categorized as a high production volume chemical in USA. Only two manufacturers in the world synthesize these compounds: OxyChem, with a current annual production of 450–4500 tons, and Anpo Electrochemical Co. (Jiangsu, China) with a volume production of 300–1000 tons/year [6].

DP and related compounds are persistent in the environment, subject to long-range atmospheric transport, biomagnification and bioaccumulation in biota through the food chain. DP’s estimated half-life in water is more than 24 years, with minimal or no anaerobic degradation [5]. It has been observed worldwide in air and marine environment from the Arctic to the Antarctic, indicating its long-range atmospheric transport potential [7]. DRCs are ubiquitous substances worldwide due to the characteristics listed above and have been reported in different matrices. The first detection of DP was reported in 2006 in the Great Lakes basin in North America [8] in air, fish and sediments samples. Following this, other studies have been carried out over the years highlighting the presence of DP in environmental matrices like air, water, soil and sediment [7,9,10,11,12,13,14,15]. Its presence in wild animals such as fish [9,12,14,16,17,18,19,20,21], birds [14,22,23] and mammals [14,24,25] confirms its biomagnification potential. DP was also detected in human samples such as serum, breast milk, hair, blood and adipose tissue [1,26,27,28,29]. This highlights how the DRCs contamination is widespread at a geographical and biota level.

Human toxicity data are still limited, but toxicological research indicated that oral exposure to DP can induce hepatic oxidative damage, alterations of metabolism and signal transduction in male mice [30]. According to Barón et al. [31], DP is genotoxic to mussels, and Chen et al. [32] highlighted how DP exposure causes neurobehavioral anomalies and potential endocrine disruption in zebrafish. This makes this compound a concern for public safety [33].

Due to these features, DP has been classified by the European Chemicals Agency (ECHA) into the Candidate List of Substances of Very High Concern and is currently under review to become part of the substances listed in the Stockholm Convention having been determined that DP isomers meet the screening criteria specified in Annex D [34].

Human exposure to DRCs has been shown to be possible through air inhalation [35], dust ingestion [36] and dietary intake [37,38,39,40]. Perinatal exposure through the mother’s blood and milk is also possible [34].

Toxicity studies in experimental animals suggest low concern for acute toxicity via oral, inhalation and dermal routes of exposure. However, there are some data gaps, for example, there are no long-term studies exceeding 90 days, which might be important given the apparently slow uptake of the substance. Therefore, toxicity testing has been required by ECHA [34]. As a result, there are currently insufficient elements to define human Tolerable Daily Intake (TDI) for DRCs and much less maximum residue limits (MRLs) have been defined in food. Studies on the presence of DRCs in foodstuff matrices are also important to determine the level of exposure of the population, providing adequate data for the risk assessment. The purpose of this review is to report the worldwide contamination levels of DRCs measured in various categories of food. This is done by comparing the literature currently available and highlighting gaps and research needs with respect to the state of the art.

## 2. Literature Search and Data Management

A thorough literature search was conducted using several electronic bibliographic databases with different keywords such as “Dechlorane” OR “Flame retardants” AND “food” OR “fish”.

At present there are still few publications reporting data on DRC food contamination. In this review, only data on food are considered. Data on food supplements, animals or parts thereof not used as food were not included. In particular, for fish and seafood, both the work on dietary intake of DCRs and those carried out on edible species for environmental monitoring purposes were considered.

The tables report data on the most evaluated DRCs in the literature such as Mirex, Dec-601, Dec-602, Dec-603, Dec-604, CP, DP-*syn* and DP-*anti*.

For the calculation, raw data present in the supplementary files, if available, were used, excluding the values not consistent with the criteria described above. The data have been reported and standardized in tables in order to facilitate comparison. A column shows the country where the food was collected even if this does not always correspond to the place of production.

The units of the measured concentrations have all been converted to pg g^−1^ (a ”×10³” has been added to the values which were expressed in ng g^−1^). Furthermore, many works express concentration values in picogram on gram of wet weight (pg g^−1^ ww), while some report them in picogram on gram of lipid weight (pg g^−1^ lw); where possible, both values are shown in the tables. These differences make comparison difficult given the various lipid content in foods.

Some authors use the lower bound (LB) and/or upper bound (UB) approach by analyzing data for left-censored values (results below the limit of detection-LOD or below the limit of quantification-LOQ). These data, if reported, have also been included in the tables, bearing in mind that the methods of calculating the LB and UB are not always standardized among the different works.

Again, to facilitate comparison, where possible, the foods have been grouped into macro-categories (e.g., meat and meat products) reporting the resulting average values.

**Figure 1:**
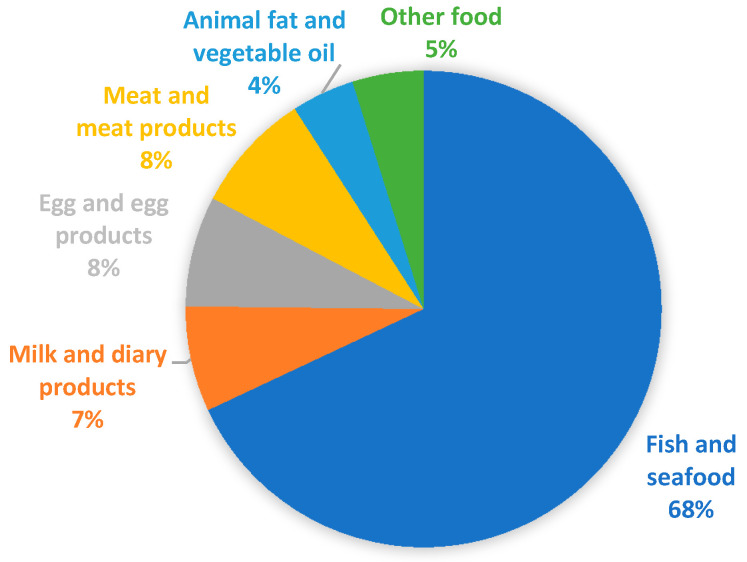
Percentage distribution among the various food categories of the number of samples examined in the articles consulted.

Figure 1 represents the percentage distribution of samples analyzed in the various food categories. As is evident, data is strongly unbalanced on fish and seafood (970 equal to 68%). For this reason, more details are provided in Table 2 and a specific section has been dedicated as the available data are far greater than for other food categories. For the other categories of food of animal origin there is a fair distribution: milk and milk products (102 equal to 7%); egg and egg products (107 equal to 8%); meat and meat products (117 equal to 8%). For the other food categories, the values are lower: animal fat and vegetable oil (61 equal to 4%); other food, mainly represented by vegetables (69 equal to 5%).

The fractional abundance of the *anti*-isomer (*f*_anti_) calculated by dividing the concentration of *anti*-DP by the sum concentration of *syn-* and *anti*-DP is also reported and discussed.

## 3. Reported Levels

Table 2 shows data on the concentration of DRCs in fish and seafood, Table 3 in milk and dairy products, egg and egg products and meat and meat products and Table 4 in animal fat and vegetable oil and other foods.

Fish and seafood represent one of the main sources of exposure to environmental contaminants for the general population [41,42]. The greater availability of information on DRCs contamination of aquatic organisms in literature is explained by the fact that these data are often collected to monitor pollution levels in aquatic ecosystems. Therefore, the works collected include those carried out for environmental monitoring purposes [9,12,14,16,17,18,19,20,21,43,44,45,46] and those carried out on food products to estimate the dietary intake of DRCs [37,38,39,40,45,47,48,49,50,51,52,53].

Some papers report DRC levels much higher than others as in the case of fishes from a highly contaminated site in China [12] and poultry and eggs collected near e-waste treatment areas in Vietnam and China [52,53], confirming the effect of this type of activity on human exposure [52].

DP, expressed as the sum of *syn* and *anti*-isomers (∑DP), has been the most studied among all the DRCs. In addition to Mirex, the DRCs most investigated are Dec-602, Dec-603, CP. Dec-601 was only investigated in three papers [20,49,51]. Dec-604 was mainly investigated in fish and seafood.

### 3.1. Concentrations of ƩDP in Fish and Seafood

Among all the DRCs, DP was the more frequently quantified compound in all the papers examined, with the only exceptions in the two works by Poma et al. [50,54] and in that of Tao et al. [52]. As shown in Table 2, apart from data obtained in samples collected near electronic waste treatment areas [12], the highest concentrations of ∑DP (223.21 × 10³ pg g^−1^ lw) were reported by Aznar-Alemany et al. [48] in commercial seafood available in European markets. The average, recalculated considering only raw samples, is strongly influenced by the presence of a highly contaminated sample of mussels collected in Denmark. Other high levels (in the order of ng g^−1^ lw) have been found in various studies on freshwater fish in Europe [17,20,21,44,46], in Korea [16], and in various Korean seafood (especially shellfish) [38].

The data obtained by expressing the concentrations in ww obviously appear lower and also included in a smaller range (from a few units to a few tens of ng g^−1^).

Alongside the non-homogeneity of the data, there are various elements that make the comparison of data on fish and seafood complex. This could be attributed the phylogenetic variety of aquatic animals with consequent different position in the food chain, metabolism, lipid content, habitat (marine or freshwater) and production methods (fished or farmed). The widespread DP contamination in fish and seafood is evidence of its global distribution both in marine and freshwater environment.

### 3.2. Concentrations of ƩDP in Other Food Categories

Again, among all DCRs, DP was the most present contaminant in the considered works, excluding the samples of milk and dairy products reported by Abdel Malak et al. (2019) [49] where the main contaminant was Dec-602. Among all the categories of other foods, eggs and egg products were on average the most contaminated.

In milk and dairy products, the highest average concentrations have been measured in Korea (23.87 pg g^−1^ ww; 928.52 pg g^−1^ lw) [38] and in Latvia (16.41 pg g^−1^ ww) [40]. Lower values of ∑DP were found in Lebanon [49], in Belgium [39,50], in the sub-Saharan countries [51], while it was not quantified in Japan [37] and in one sample from Belgium [54].

Apart from data obtained in samples collected near electronic waste treatment areas, the highest mean concentration of ∑DP in all categories of foodstuffs has been observed in Chinese chicken eggs with a value of 123.6 × 10³ pg g^−1^ lw [53]. These egg samples had been collected in southern China and used as a reference value to compare with that of contaminated areas. The second highest value in egg and egg products is one reported in Belgium (159 pg g^−1^ ww) [50]. In particular ∑DP was measured only in one out of the four analyzed samples (quail eggs 637 pg g^−1^ ww) [50]. Lower values (with a range from 1.27 to 30.33 pg g^−1^ ww) were measured in samples from Lebanon [49], Japan [37], Korea [38], Belgium [39], sub-Saharan countries [51] and Latvia [40]. It is well known that eggs can be a good environmental indicator of persistent organic contamination [38]. This is why some studies have focused on DP contamination in wild bird eggs. In the latter, the levels measured are usually higher than those measured in chicken eggs. The reason may be that the body burden in wild birds is high due to slower depuration because fewer eggs are laid [38], possibly higher longevity and/or higher trophic level.

In meat and meat products, DP contamination reproduce a trend similar to that of milk and milk products. Again, excluding data obtained in electronic waste treatment areas in Vietnam and China [48,49], the highest mean concentrations of ∑DP were measured in Korea (51.86 pg g^−1^ ww; 959.36 pg g^−1^ lw) [38], considering that this result is reported according to an LB scenario. Lower values (with a range from 1.5 to 20.13 pg g^−1^ ww) were measured in samples collected in Lebanon [49], Japan [37], Belgium [50], sub-Saharan countries [51] and Latvia [40]. Other studies, conducted on a small number of samples, gave negative results [52,54].

Concerning vegetable oils and animal fat, the mean concentrations show a tighter range (1.53–21.1 pg g^−1^ ww LB and 11.13–52.8 pg g^−1^ ww UB). In Japan [37] and Belgium [50] DP has not been quantified.

Some authors reported data on DP contamination in food of vegetable origin [37,38,40,50,51,54] In Table 4 only samples with DP levels above zero are reported. The ∑DP levels were not detected in biscuit and potato chips [54], in grain and grain products, potatoes and derived products [50], and rice and rice products, grain, seed, tubers, fruits, vegetable, seasoning and other processed food [37]. In studies conducted in Korea [38], sub-Saharan countries [51] and Latvia [40] most of the samples examined showed quantifiable levels of DP with the highest values measured in noodle (50.16 pg g^−1^ ww). The study conducted in Japan showed detectable traces of DP only in samples of sugar and confectionery, legumes and their products (3.3 and 2.8 pg g^−1^ ww respectively) [37].

### 3.3. Profiles of DP Isomers

In commercial products the two isomers are present in a ratio of about 3:1, so the enantiomeric fraction of *anti*-DP (*f*_anti_), defined as the concentration ratio of *anti*-DP over ∑DP, presents a value from 0.65 to 0.80. This ratio is generally used to assess environmental fate and distribution of DP. In biota, different studies report values of *f*_anti_ at around 0.6 depending on the origin of the matrix, due to different *syn*-DP enrichments in the environment [49]. In Table 2, Table 3 and Table 4 the values of *f*_anti_ have been calculated for all samples when DP contamination was quantified; these values show a great variability, ranging from 0.13 in freshwater fish [18] to 1.00 in fish [14], milk, egg and oil and fat samples [51]

In L’Homme et al. [39] the *f*_anti_ was about 0.3. This value is affected by the fact that the LOQs obtained in the various matrices for *syn*-DP (ranging from 1.49–20.00 pg g^−1^) were a little higher than those of *anti*-DP (ranging from 0.52–5.42 pg g^−1^). Therefore, using the UB scenario, apparently higher *syn*-DP levels were reported, considering that most of the samples analyzed were below the LOQ [36]. Hence it is evident that the different analytical limits related to *syn*-DP and *anti*-DP can greatly influence the *f*_anti_ value.

Among the articles considered, the values of *f*_anti_ in fish and seafood have a great variability with a range between 0.13 and 1 (average value of 0.57). Very low values (0.13 and 0.23) have been reported in freshwater fishes in the two works of Sühring et al. [17,18]. The highest values of *f*_anti_ in this food category were 1 [14] in sampled fishes in USA. This great variability in the ratio values would not only depend on the different levels of environmental contamination, but also on the differences in metabolism between the various aquatic organisms. [17]. The average value of *f*_anti_, closed to 0.60, obtained in fish and seafood category would indicate a tendency to the bioaccumulation and biomagnification of *syn*-DP compared to *anti*-DP [12,17].

The lowest average values of *f*_anti_, in other food categories were observed in milk and dairy products and in meat and meat products (0.65 and 0.64 (LB) respectively), suggesting a significant enrichment in *syn*-DP due either to mammalian metabolic processes and/or to technological processes such as fermentation [49].

In the other food items, the average values of *f*_anti_ were higher (egg = 0.72, animal and vegetable fat = 0.76, other food = 0.70, (LB values)) suggesting that the contamination could have occurred by direct contact with the technical product, especially for vegetable products, and in any case without biological enrichment process.

### 3.4. Concentration of Other DRCs in Fish and Seafood Products

The most frequently quantified DRC after DP is Dec-602, with the highest concentrations found in freshwater fishes in Spain (52.2 × 10³ pg g^−1^ lw) [44]. Other values, significantly lower, but still of the order of ng g g^−1^ lw were again measured in freshwater fish from Italy [46] and in eel from Germany [17]. From the analysis of the data, it emerges that Dec-602, where it was quantified, shows much higher levels than the ∑DP in most samples.

The highest mean value of Dec-603 (11.35 × 10³ pg g^−1^ lw) was detected by Aznar-Alemany et al. [48] in fish and seafood from the European market. Again, the mean value, recalculated considering only raw samples, is affected by the presence of highly contaminated samples of mussels collected in Italy and sea bream of undeclared origin. Values slightly higher than 2 × 10³ pg g^−1^ lw were also found in freshwater fish from Spain [44]. In almost all cases there is a simultaneous presence of Dec-602 and Dec-603 (often in smaller quantities).

Dec-604 was quantified in two works. The highest contamination level (2.07 × 10³ pg g^−1^ lw) was measured in freshwater fish in Italy [46]. The other quantifiable sample (0.288 × 10³ pg g^−1^ lw) was obtained in a sample of mussel collected in Denmark [48].

CP was quantified in four works. The highest concentration (100 pg g^−1^ lw LB) was detected in samples of catfish from France [20], followed by samples of salmon collected in Belgium [39] with a mean contamination level of 4.24 pg g^−1^ lw.

Quantifiable level of Mirex were reported in five papers dealing with DRCs. A rather high average value (11.10 × 10³ pg g^−1^ lw) was measured in Baltic salmon from Latvia [19]. Another Latvian work quantified a lower mean level of contamination of 60 pg g^−1^ lw in European eels [14]. L’Homme et al. [39], reported a mean value of 15.53 pg g^−1^ lw in salmon. The other two work report a concentration expressed in the ratio ww of Mirex just over 20 pg g^−1^ in Latvian [40] and Korean [38] samples.

Among the papers selected, Dec-601 was evaluated in samples from France [20], Lebanon [49] and sub-Saharan countries [51], with lower values than the other DRCs (<1 pg g^−1^ ww).

### 3.5. Concentration of Other DRCs in Other Food Categories

For this category, five papers among those consulted take into consideration the presence of other DRCs in addition to the DP [32,33,40,49,51]. Also, in this case Dec-602 and Dec-603 were the most often quantified alternative compounds to DP.

In milk and dairy products, the highest levels of Dec-602 contamination, with similar mean values of about 3 pg g^−1^ ww, were measured in Lebanon [49] and Latvia [40]. Dec-603 mean values are also higher in the study conducted in Latvia (2.39–2.80 pg g^−1^ ww LB-UB) [40]. CP contamination in milk and dairy products is very low. The three papers [38,39,40] that evaluated Mirex give substantially uniform average values in the range from 0.50 to 0.88 pg g^−1^ ww. Dec-601, evaluated in two papers [49,51], never exceeded the LOQs of the methods used.

In egg and egg products the highest values of Dec-602 (1.2–1.7 pg g^−1^ ww LB-UB) and CP (1.36–1.41 pg g^−1^ ww LB-UB) are those found in the study conducted in Lebanon [49], while highest value Dec-603 was measured in one sample from Cameroon [51]. For Dec-601 no values higher than the LOQ were obtained. Mirex was detected at low concentrations in samples from Belgium [39] and Latvia [40].

In meat and meat products the values of these DRCs are generally low. The highest levels are those of Dec-602 (3.54 pg g^−1^ ww) in a Korean study [38] and (up to 1.80 pg g^−1^ ww) in the sub-Saharan data [51].

In the Korean study [38], in addition to Dec.602, Mirex was detected (1.71 pg g^−1^ ww), despite this compound was not registered in that country as a pesticide. The authors suggested that the environment around Korea may have been influenced by a long-range transport of Mirex from China, where it was used as insecticide.

In animal fat and vegetable oil Dec-602 is the compound that gave the highest mean value among the other DRCs in Lebanese vegetable oil (3.0–11.8 pg g^−1^ ww LB-UB) [49], followed by data from sub-Saharan countries (from 1.5 to 3.0 pg g^−1^ ww) [51]. The highest mean value of Dec-603 was recorded in samples from Benin (4.5–6.6 pg g^−1^ ww LB-UB). The greatest contamination of CP was measured in Lebanon (3.2–3.7 pg g^−1^ ww LB-UB) [49], Mirex showed the higher value (0.43 pg g^−1^ ww) in animal fat from Belgium [39]. Dec-601 was always below the LOQ.

Finally, in the “other foods” category, DRCs were quantified only in two studies: one in bread and cereals in Latvia [40] one in products from on sub-Saharan countries [51].

In the first work, both Dec-602 (3.83–3.86 pg g^−1^ ww LB-UB) and Dec-603 (3.18–3.26 pg g^−1^ ww LB-UB) showed the highest values. CP and Mirex were only quantified in nuts and seeds in Mali [51] and in bread and cereals in Latvia [40].

## 4. Conclusions

The concern about DRCs’ environmental and biota contamination is relatively recent since the first detection was only reported in 2006 by Hoh et al. [8]. Therefore, the specific research dedicated to the presence of these contaminants in food is currently still very limited. The category of foods that is most often taken into consideration is fish and seafood. This is because these organisms are often used for monitoring both marine and freshwater aquatic ecosystems. The other food categories are often considered in studies on DRC dietary intake referring to a population of a specific country. For this reason, the various foods are often grouped into large categories, and this complicates the comparison between different monitoring. Although the production of these substances is limited to a few sites in the world, the data analysis shows that the contamination of DRCs is a global reality. The comparison of the data highlights the significant impact of the electronic waste treatment areas on DRCs environmental diffusion. Of course, all of these elements have an effect on the contamination of the food chain.

From the analysis of the various articles, it emerges that, among all the DRCs, the DP is the substance most often investigated and quantified with values that would indicate slightly higher contamination levels in fish and seafood category. However, further studies should be conducted to confirm this trend. Data on other DRCs are still limited. In addition to this, a further element that complicates the comparison is that the concentrations are not always expressed in the same way, referring the values to the lipid weight and/or wet weight.

Kim et al. [38] reports that daily intake of ΣDP in the Korean population (evaluated by analyzing 175 samples of 35 different food products from the retail market) was estimated at 11.2 10³ pg/day, and this value was 3 times higher than that calculated for the other DRCs. Similar values, referring to the sum of all DRCs, are reported in a recent work by Zacs [40] that estimated the daily intake in Latvia of 460 pg kg bw. The value multiplied by the average weight for the general population (72 kg) gives the value of 33 10^3^ pg day. In a Lebanese study on 58 samples representing fatty food groups, the estimated daily dietary intake for the adult population (25–54 years) had values between 2629 and 3922 pg/day for ΣDP (LB-UB) [49]. The dietary intake of the sum of Mirex, Dec 602, Dec 603, CP, *syn*-DP and *anti*-DP calculated by L’Homme et al. [39] in Belgium is much lower, with an average value of 136 pg/days. This could be explained by a lower presence of fatty foods among the samples considered. In conclusion, a Japanese study on a market basket of 123 food samples estimated a ΣDP daily intake for adult population of 576 pg/day [37].

To date, no work has yet evaluated and compared the daily dietary intake of these substances by considering specific populations such as any more sensitive (e.g., children) or exposed subpopulations.

The aim of this review was to provide an overview, based on available data, on the extent of DCR contamination in terms of compounds, concentration levels and different food categories. At the same time, we also wanted to highlight the difficulties faced in comparing the data due to the heterogeneity of the studies (different approaches, matrices, analytical performances, etc.). From these considerations emerge, in our opinion, the following needs to improve and standardize the study of dietary exposure to DCRs. In addition to the DP, other DRCs must be included in the surveys, in line with the need to evaluate the potential “combined effects” of chemical mixtures in food. Uniform methods of expression of results should be favored, which also take into account the lipid percentage of food. Data on foods other than fish and seafood should be increased by carefully distinguishing the various categories of foodstuff. The geographical origin of food should be better defined to have a general and equally distributed picture of worldwide contamination.

This could be achieved in the structured framework of total diet studies (TDS) to complement toxicological studies establishing reference values (e.g., TDI) necessary to perform a correct risk assessment of these compounds in foods and within their supply chain.

## Figures and Tables

**Table 1:** Molecular formula, abbreviations and structural formula of Mirex, Dechlorane Plus and other DRCs.

Compounds	Abbreviations	Molecular Formula	Chemical Structure
Mirex		C_10_Cl_12_	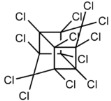
Dechlorane Plus	DP or DDC-CO	C_18_H_12_Cl_12_	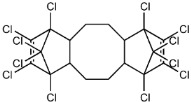
Dechlorane 601	Dec-601 or DDC-ID	C_20_H_12_Cl_12_	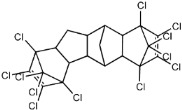
Dechlorane 602	Dec-602 or DDC-DBF	C_14_H_4_Cl_12_O	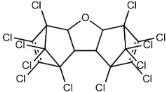
Dechlorane 603	Dec-603 or DDC-Ant	C_17_H_8_Cl_12_	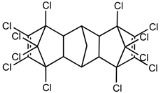
Dechlorane 604	Dec-604 or HCTBPH	C_13_H_4_Br4C_l6_	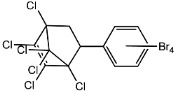
Chlordene Plus	CP or DDC-PDD	C_15_H_6_C_l12_	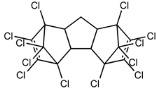

**Table 2:** Comparison of data on DRCs concentration in fish and seafood reported in different studies (data are expressed as mean value in pg g^−1^).

Authors, Year, [Reference]	Country	Unit	Scenario	Fish	*Mirex*	*Dec-601*	*Dec-602*	*Dec-603*	*Dec-604*	*CP*	*syn-DP*	*anti-DP*	*Ʃ* *DP*	*f_anti_*
Abdel Malak et al., 2018 [20]	France	ww (lw)	LB-UB	Catfish (*n* = 102)	-	ND	11.8–11.8 (555–555)	11.9–11.9 (499–501)	-	2.24–2.25 (100–102)	2.60–4.60 (189–506)	5.45–7.04 (370–637)	8.05–11.64 (559–1143)	0.67–0.60 (ww)
Abdel Malak et al., 2019 [36]	Lebanon	ww	LB-UB	Fish (*n* = 21)	-	0.0–0.3	7.0–7.8	0.2–0.3	-	0.4–0.5	2.0–4.2	3.0–3.9	5.0–8.0	0.60–0.48
Aznar-Alemany et al., 2017 [48]	Europe	lw	UB	Fish and seafood (*n* = 42)	-	-	70	11.35 × 10³	90	-	63.78 × 10³	159.43 × 10³	223.21 × 10³	0.71
Giulivo et al., 2017 [46]	Greece	lw	UB	Freshwater fish (*n* = 4)	-	-	ND	ND	ND	-	ND	ND	-	-
	Slovenia Croatia Bosnia-Herzegovina Serbia			Freshwater fish (*n* = 10)	-	-	ND	ND	ND	-	510	770	1.28 × 10³	0.60
	Italy			Freshwater fish (*n* = 13)	-	-	2.60 × 10³	ND	2.07 × 10³	-	ND	ND	-	-
Houde et al., 2014 [45]	Canada	lw	-	Yellow perch (*n* = 29)	-	-	ND	ND	ND	ND	ND	ND	ND	-
Kakimoto et al., 2012 [47]	Japan	ww	LB	Saltwater fish (*n* = 20)	-	-	-	-	-	-	0.83	1.39	2.22	0.62
Kakimoto et al., 2014 [37]	Japan	ww	-	Fish, shellfish, their products (*n* = 17)	-	-	-	-	-	-	1.0	0.9	1.9	0.49
Kang et al., 2010 [16]	Korea	lw	-	Freshwater fish (*n* = 22)	-	-	-	-	-	-	8.1 × 10³	16.9 × 10³	25.0 × 10³	0.68
Kim et al., 2014 [38]	Korea	ww (lw)	LB	Fish and shellfish (*n* = 70)	26.33 (460.44)	-	3.99 (166.04)	ND (0.55)	-	-	8.25 (316.33)	28.09 (1031.95)	36.34 (1348.28)	0.77 (ww)
Klosterhaus et al., 2012 [14]	USA	lw		Fish (*n* = 14)	-	-	-	-	-	-	ND	957	957	1.00
L’Homme et al., 2015 [39]	Belgium	lw	UB	Salmon (*n* = 8)	15.53	-	1.75	3.72	-	4.24	4.24	1.89	6.13	0.30
Poma et al., 2016 [54]	Belgium	ww	LB	Fish and fish products (*n*= 11)	-	-	-	-	-	-	ND	ND	ND	-
Poma et al., 2018 [50]	Belgium	ww	LB	Fish and fish products (*n*= 61)	-	-	-	-	-	-	ND	ND	ND	-
Ren et al., 2013 [43]	China	lw	-	River fish (*n* = 149)	-	-	-	-	-	-	82	141	223	0.63
Rjabova et al., 2016 [19]	Latvia	lw	-	Baltic salmon (*n* = 25)	11.10 × 10³	-	370.0	36.4	ND	-	85.6	159.0	244.6	0.65
Santín et al., 2013 [44]	Spain	lw	-	Freshwater fish (*n* = 48)	-	-	52.2 × 10³	2.6 × 10³	-	-	520	620	1.14 × 10³	0.54
Sühring et al., 2013 [17]	Germany	ww (lw)	-	European eel (*n* = 45)	-	-	600 (1.17 × 10³)	ND (10)	ND	-	20 (590)	10 (180)	30 (770)	0.23 (0.29)
Sühring et al., 2016 [18]	Germany	ww	-	Freshwater fish (*n* = 44)	-	-	77	ND	ND	ND	20	3	23	0.13
Tao et al., 2016 [52]	Vietnam	lw	-	River fish (*n* = 5)	-	-	-	-	-	-	ND	ND	ND	-
Tomy et al., 2007 [9]	Canada	lw		Freshwater fish (*n* = 44)	-	-	-	-	-	-	183	259	442	0.59
Vaccher et al., 2020 [51]	Cameroon	ww	LB-UB	Fish (*n* = 4)	-	0.00–0.37	6.34–6.64	0.21–0.64	-	0.51–0.52	0.89–1.59	1.00–1.31	1.89–2.90	0.52–0.45
	Mali			Fish (*n* = 2)	-	0.00–0.80	27.55–27.55	1.32–1.33	-	1.15–1.16	5.68–7.87	10.19–11.26	15.87–19.13	0.64–0.58
	Benin			Fish (*n* = 2)	-	0.00–1.03	4.37–4.38	0.71–0.88	-	0.69–0.71	3.68–5.55	5.88–7.25	9.56–12.80	0.61–0.57
	Nigeria			Fish (*n* = 1)	-	0.00–0.10	11.07–11.07	0.00–0.38	-	0.46–0.46	0.69–0.87	1.23–1.31	1.92–2.18	0.64–0.60
Wu et al., 2010 [12]	China	lw		Freshwater fish (*n* = 86)	-	-	-	-	-	-	119.9 × 10³	219.8 × 10³	339.7 × 10³	0.65
				Control freshwater fish (*n* = 5)	-	-	-	-	-	-	1.4 × 10³	7.4 × 10³	8.8 × 10³	0.85
Zacs et al., 2018 [14]	Latvia	lw	LB	European eel (*n* = 58)	60	-	250	10	ND	-	60	200	260	0.76
Zacs et al., 2021 [40]	Latvia	ww	LB-UB	Fish (*n* = 8)	20.63–20.64	-	15.94–16.16	18.24–18.78	-	-	5.01–5.01	9.45–9.45	14.46–14.46	0.65–0.65

“ND” = values below the limit of detection are labeled “Not Detected” (ND); “-“ = not included in the study.

**Table 3:** Comparison of data on DRCs concentration in milk and dairy products, egg and egg products and meat and meat products reported in different studies (data are expressed as mean value in pg g−1).

Authors, Year [Reference]	Country	Unit	Scenario	Food	*Mirex*	*Dec-601*	*Dec-602*	*Dec-603*	*CP*	*syn-DP*	*anti-DP*	*Ʃ* *DP*	*f_anti_*
**Milk and dairy products**													
Abdel Malak et al., 2019 [49]	Lebanon	ww	LB-UB	Milk and dairy products (*n* = 13)	-	0.0–0.1	2.9–3.3	0.05–0.1	0.01–0.03	1.0–2.0	0.7–1.1	1.7–3.1	0.41–0.35
Kakimoto et al., 2014 [37]	Japan	ww	-	Milk and dairy products (*n* = 5)	-	-	-	-	-	ND	ND	ND	-
Kim et al., 2014 [38]	Korea	ww (lw)	LB	Milk and dairy products (*n* = 15)	0.82 (36.68)	-	ND	ND	-	4.42 (173.78)	19.45 (754.74)	23.87 (928.52)	0.81
L’Homme et al., 2015 [39]	Belgium	lw	UB	Milk (*n* = 16)	0.50	-	0.89	1.06	0.26	12.50	5.11	17.61	0.30
Poma et al., 2016 [54]	Belgium	ww	LB	Milk (*n* = 1)	-	-	-	-	-	ND	ND	ND	-
Poma et al., 2018 [50]	Belgium	ww	LB	Milk and dairy products (*n* = 38)	-	-	-	-	-	2	7	9	0.78
Vaccher et al., 2020 [51]	Cameroon	ww	LB-UB	Milk and dairy products (*n* = 1)	-	0.00–0.84	0.28–0.57	0.00–1.24	0.00–0.09	0.00–1.30	0.25–0.89	0.25–2.19	1–0.40
	Mali			Milk and dairy products (*n* = 2)	-	0.00–0.18	0.31–0.31	0.00–0.16	0.02–0.04	0.21–0.98	0.39–0.90	0.60–1.88	0.65–0.48
	Benin			Milk and dairy products (*n* = 3)	-	0.00–0.39	0.17–0.17	0.00–0.31	0.00–0.02	0.07–0.75	0.06–0.69	0.13–1.44	0.46–0.48
	Nigeria			Milk and dairy products (*n* = 1)	-	0.00–0.94	0.66- 0.66	0.00–0.66	0.00–0.13	0.66–1.29	2.18–2.46	2.84–3.75	0.76–0.66
Zacs et al., 2021 [40]	Latvia	ww	LB-UB	Milk and dairy products (*n* = 8)	0.88–0.88	-	3.44–3.60	2.39–2.80	-	5.60–5.60	10.81–10.81	16.41–16.41	0.66–0.66
**Egg and egg products**													
Abdel Malak et al., 2019 [49]	Lebanon	ww	LB-UB	Egg (*n* = 5)	-	0.0–0.3	1.2–1.7	0.2–0.4	1.36–1.41	1.7–3.1	5.2–5.8	6.9–8.9	0.75–0.65
Kakimoto et al., 2014 [37]	Japan	ww	-	Meat and eggs (*n* = 13)	-	-	-	-	-	0.6	0.9	1.5	0.60
Kim et al., 2014 [38]	Korea	ww (lw)	LB	Egg (*n* = 5)	ND	-	ND	ND	-	3.19 (17.64)	12.12 (67.32)	15.31 (84.96)	0.79
L’Homme et al., 2015 [39]	Belgium	lw	UB	Egg (*n* = 8)	0.21	-	1.28	2.76	0.94	20.00	6.27	26.27	0.24
Poma et al., 2016 [54]	Belgium	ww	LB	Egg (*n* = 2)	-	-	-	-	-	ND	ND	ND	-
Poma et al., 2018 [50]	Belgium	ww	LB	Egg and egg products (*n* = 4)	-	-	-	-		32	127	159	0.80
Tao et al., 2016 [52]	Vietnam	lw	-	Chicken egg (*n* = 15)	-	-	-	-	-	140 × 10³	450 × 10³	590 × 10³	0.76
	Vietnam Japan	lw	-	Control chicken egg (*n* = 2)	-	-	-	-	-	ND	ND	ND	-
Vaccher et al., 2020 [51]	Cameroon	ww	LB-UB	Eggs (*n* = 1)	-	0.00–0.06	0.57–0.65	1.80–1.80	0.00–0.01	2.02–2.45	6.46–6.64	8.48–9.09	0.76–0.73
	Mali			Eggs (*n* = 1)	-	0.00–0.26	0.79–0.79	0.00–0.31	0.00–0.05	0.00–0.93	1.27–1.74	1.27–2.67	1.00–0.65
	Benin			Eggs (*n* = 1)	-	0.00–0.06	0.00–0.01	0.29–0.29	0.00–0.02	1.28–1.67	3.50–3.78	4.78–5.45	0.73–0.69
	Nigeria			Eggs (*n* = 1)	-	0.00–0.38	1.15–1.15	0.00–0.50	0.00–0.08	1.82–2.00	4.24–4.32	6.06–6.32	0.70–0.68
Zacs et al., 2021 [40]	Latvia	ww	LB-UB	Eggs (*n* = 8)	0.00–1.27	-	0.71–0.59	0.39–0.60	-	8.03–8.03	22.31–22.31	30.33–30.33	0.74–0.74
Zheng et al., 2012 [53]	China	lw		Chicken egg (*n* = 33)	-	-	-	-	-	407 × 10³	1192 × 10³	1599 × 10³	0.75
				Control chicken egg (*n* = 8)	-	-	-	-	-	28 × 10³	95.6 × 10³	123.6 × 10³	0.77
**Meat and meat products**													
Abdel Malak et al., 2019 [49]	Lebanon	ww	LB-UB	Meat and poultry (*n* = 12)	-	0.0–0.2	0.0–0.7	0.1–0.3	0.0–0.05	10.1–1.8	7.4–8.1	17.4–19.9	0.43–0.41
Kakimoto et al., 2014 [37]	Japan	ww	-	Meat and eggs (*n* = 13)	-	-	-	-	-	0.6	0.9	1.5	0.60
Kim et al., 2014 [38]	Korea	ww (lw)	LB	Meat and meat products (*n* = 35)	1.71 (11.12)	-	3.54 (21.55)	ND	-	11.61 (234.74)	40.25 (724.62)	51.86 (959.36)	0.78 (ww)
L’Homme et al., 2015 [39]	Belgium	lw	UB	Meat and poultry (*n* = 16)	0.16	-	0.43	0.20	0.08	7.14	2.88	10.02	0.28
Poma et al., 2016 [54]	Belgium	ww	LB	Meat and meat products (*n* = 1)	-	-	-	-	-	ND	ND	ND	-
Poma et al., 2018 [50]	Belgium	ww	LB	Meat and poultry (*n* = 3)	-	-	-	-	-	2	8	10	0.80
Tao et al., 2016 [52]	Vietnam	lw	-	Chicken (*n* = 15)	-	-	-	-		693 × 10³	1683 × 10³	2376 × 10³	0.71
	Vietnam	lw	-	Pork (*n* = 2)						ND	ND	ND	-
	Vietnam Japan	lw	-	Control chicken (*n* = 4)						ND	ND	ND	-
	Vietnam	lw	-	Control pork (*n* = 1)						ND	ND	ND	-
Vaccher et al., 2020 [51]	Cameroon	ww	LB-UB	Meat (*n* = 2)	-	0.00–0.20	0.80–0.93	0.00–0.75	0.11–0.12	6.09–6.73	14.04–14.72	20.13–21.45	0.70–0.69
	Mali			Meat (*n* = 1)	-	0.00–0.12	1.80–1.80	0.00–0.42	0.00–0.01	1.66–3.16	2.10–2.83	3.76–5.99	0.56–0.47
	Benin			Meat (*n* = 2)	-	0.00–0.15	1.56–1.56	0.00–0.24	0.00–0.02	0.57–1.19	1.61–2.07	2.18–3.26	0.74–0.63
	Nigeria			Meat (*n* = 2)	-	0.00–0.25	1.41–1.41	0.00–0.88	0.00–0.04	2.02–2.26	4.29–4.39	6.31–6.65	0.70–0.66
Zacs et al., 2021 [40]	Latvia	ww	LB-UB	Meat (*n* = 8)	0.03–0.58	-	0.67–1.38	0.59–1.43	-	2.64–2.64	5.88–5.88	8.52–8.52	0.69–0.69

ND” = values below the limit of detection are labeled “Not Detected” (ND); “-“ = not included in the study.

**Table 4:** Comparison of data on DRCs concentration in animal fat and vegetable oil and other food reported in different studies (data are expressed as mean value in pg g^−1^).

Authors, Year [Reference]	Country	Unit	Scenario	Food	*Mirex*	*Dec-601*	*Dec-602*	*Dec-603*	*CP*	*syn-DP*	*anti-DP*	*Ʃ* *DP*	*f_anti_*
**Animal and Vegetable fat**													
Abdel Malak et al., 2019 [49]	Lebanon	ww	LB-UB	Vegetable oil (*n* = 7)	-	0.0–4.6	3.0–11.8	2.3–3.9	3.2–3.7	2.4–25.0	18.7–27.9	21.1–52.8	0.89–0.53
Kakimoto et al., 2014 [37]	Japan	ww	-	Oils and fats (*n* = 4)	-	-	-	-	-	ND	ND	ND	-
Kim et al., 2014 [38]	Korea	ww	LB	Soy oil (*n* = 5)	ND	-	ND	ND	-	3.19	12.12	15.31	0.79
L’Homme et al., 2015 [39]	Belgium	lw	UB	Animal fat (*n* = 18)	0.43		0.75	0.57	0.16	12.50	6.60	19.10	0.35
				Vegetable oil (*n* = 2)	0.13		0.75	0.50	0.20	12.50	6.61	19.11	0.35
Poma et al., 2016 [54]	Belgium	ww	LB	Vegetable fat (*n* = 1)						ND	ND	ND	-
Poma et al., 2018 [50]	Belgium	ww	LB	Animal and Vegetable fat (*n* = 9)						ND	ND	ND	-
Vaccher et al., 2020 [51]	Cameroon	ww	LB-UB	Oil and fat (*n* = 3)	-	0.00–2.08	1.66–3.00	0.00–6.03	0.67–0.84	0.00–4.53	5.2–6.03	5.2–10.65	1.00–0.57
	Mali			Oil and fat (*n* = 2)	-	0.00–3.62	1.56–1.56	0.00–2.56	0.00–0.54	0.00–3.21	1.97–7.46	1.97–10.67	1.00–0.70
	Benin			Oil and fat (*n* = 2)	-	0.00–5.89	1.88–1.88	4.5–6.60	0.00–0.19	0.00–2.95	1.53–8.18	1.53–11.13	1.00–0.73
	Nigeria			Oil and fat (*n* = 2)	-	0.00–2.99	2..44–2.44	0.00–1.99	0.21–0.58	4.21–5.80	12.23–12.94	16.44–18.74	0.74–0.69
Zacs et al., 2021 [40]	Latvia	ww	LB-UB	Vegetable oil (*n* = 4)	0.00–0.79	-	0.31–2.02	0.00–5.76	-	2.00–4.40	5.57–10.00	7.57–14.40	0.74–0.69
**Other foods**													
Kakimoto et al., 2014 [37]	Japan	ww	-	Legumes and their products (*n* = 7)	-	-	-	-	-	0.9	1.9	2.8	0.68
			-	Sugar and confectionary (*n* = 7)	-	-	-	-	-	1.0	2.3	3.3	0.70
Kim et al., 2014 [38]	Korea	ww	LB	Vegetables (*n* = 15)	ND	-	ND	ND	-	0.42	1.86	2.28	0.82
				Grain (*n* = 5)	ND	-	ND	ND	-	2.95	18.73	21.68	0.86
				Fruit (*n* = 5)	ND	-	ND	ND	-	0.99	1.23	2.22	0.55
				Noodle (*n* = 5)	ND	-	ND	ND	-	9.33	40.83	50.16	0.81
				Seaweed (*n* = 5)	ND	-	ND	ND	-	2.82	7.11	9.94	0.72
				Legume (*n* = 5)	ND	-	ND	ND	-	3.59	21.05	24.65	0.85
				Condiment (*n* = 5)	ND	-	ND	ND	-	4.81	27.81	32.62	0.85
Vaccher et al., 2020 [51]	Nigeria	ww	LB-UB	Miscellaneous (*n* = 2)	-	0.00–1.94	0.37–0.57	0.00–4.49	0.00–0.28	7.51–8.14	21.83–22.11	29.43–30.25	0.74–0.73
	Cameroon	ww	LB-UB	Nuts and seeds (*n* = 1)	-	0.00–2.67	0.35–0.85	0.00–2.26	0.00–0.31	3.21–5.73	9.97–11.05	13.18–16.78	0.76–0.66
	Mali			Nuts and seeds (*n* = 1)	-	0.00–1.11	1.82–1.82	0.00–0.62	0.72–0.72	2.11–4.18	8.14–9.15	10.25–13.33	0.79–0.69
	Benin			Nuts and seeds (*n* = 1)	-	0.00–0.41	0.11–0.11	0.00–0.40	0.00–0.05	6.65–7.54	3.34–4.00	9.99–11.54	0.33–0.35
	Nigeria			Nuts and seeds (*n* = 1)	-	0.00–0.17	0.05–0.05	0.00–0.29	0.00–0.02	0.89–1.06	0.89–0.97	1.78–2.03	0.50–0.52
Zacs et al., 2021 [40]	Latvia	ww	LB-UB	Bread and cereals (*n* = 4)	0.96–1.00	-	3.83–3.86	3.18–3.26	-	5.97–5.97	8.35–8.50	14.32–14.40	0.58–0.59

ND” = values below the limit of detection are labeled “Not Detected” (ND); “-“ = not included in the study.

## Data Availability

No new data were created in this study. Data sharing is not applicable to this article.
